# Effects of a “5 + 2” dietary management model on body composition and behavioral modification in Chinese children with obesity: a prospective, non-randomized controlled trial

**DOI:** 10.3389/fnut.2026.1907795

**Published:** 2026-09-01

**Authors:** Huiping Su, Lili Pan, Rongfei Zheng, Chushan Wu, Yue Shang, Wei Su, Jingfan Xiong, Minlu Li, Zhe Su

**Affiliations:** 1 Department of Endocrinology, Shenzhen Children’s Hospital, Shenzhen, China; 2 Department of Child and Adolescent Chronic Disease Prevention and Control, Shenzhen Center for Chronic Disease Control, Shenzhen, China; 3 Department of Child and Adolescent Chronic Disease Prevention and Control, Longgang District Chronic Disease Prevention and Control Hospital, Shenzhen, China

**Keywords:** behavioral modification, body composition, childhood obesity, dietary intervention, high-protein diet

## Abstract

**Introduction:**

Continuous energy restriction may be associated with poor adherence and a risk of muscle mass loss in pediatric obesity management. This study evaluated the effects of a structured “5 + 2” dietary management model—cycling 5 weekdays of calorie-restricted diet with 2 weekend days of high-protein diet—supported by standardized supplements and professional supervision.

**Methods:**

In this prospective, non-randomized controlled trial conducted in Shenzhen, China, 84 children with persistent obesity (median age 11.1 years) were enrolled. The intervention group (*N* = 31) followed an 8-week “5 + 2” protocol with dietitian-led behavioral guidance and satiety-enhancing supplements, followed by a 12-week follow-up period. Controls (*N* = 53) received conventional weight-loss advice. Propensity score matching was employed to balance baseline cohorts (*N* = 74; 30 intervention, 44 control). Changes in weight, body composition, metabolic parameters, and behavior were assessed at weeks 8 and 20.

**Results:**

At week 8, the intervention group achieved significantly greater weight loss (−1.42 ± 1.73 kg vs. −0.44 ± 1.54 kg, *p* = 0.015) and body mass index (BMI) standard deviation score (SDS) reduction (−0.21 ± 0.14 vs. -0.11 ± 0.14, *p* = 0.005) compared to controls. The median behavior score in the intervention group improved significantly from 66.0 to 82.0 (*p* < 0.001), with marked increases in self-weighing frequency and physical activity. At the 20-week follow-up (post-summer break), the intervention group maintained greater reductions in body fat mass (−1.42 ± 3.41 vs. +1.84 ± 3.67 kg, *p* < 0.001) and greater skeletal muscle gain (3.90 ± 3.46 vs. 2.21 ± 2.36 kg, *p* = 0.031) than the control group, with significantly lower fat mass index (FMI) *Z*-scores and higher muscle mass index (MMI) *Z*-scores. Linear regression identified boys (*β* = −0.758, *p* = 0.015), older children (*β* = −0.240, *p* = 0.003) and children with higher baseline adherence (*β* = −0.356, *p* = 0.004) as key predictors for BMI SDS reduction.

**Conclusion:**

The “5 + 2” dietary management model was associated with favorable changes in body composition and health-related behaviors in children with obesity and may represent a practical dietary management strategy.

## 1 Introduction

Childhood obesity has become a global public health challenge. In 2022, more than 160 million children and adolescents aged 5–19 years worldwide were living with obesity, a prevalence that has tripled since 1990 (1). Childhood obesity increases the risk of adult obesity and is associated with a risk of long-term health complications, such as type 2 diabetes, cardiovascular disease, and cancer (2–4).

Nutritional management is key for the treatment of obesity. Various weight-loss diet regimens have been developed, incorporating different strategies for energy restriction, macronutrient composition, food selection, and dietary intake patterns (5, 6). According to the Chinese Guidelines for the Diagnosis and Treatment of Obesity (7, 8), the recommended dietary patterns for weight loss include a very low-energy diet, a calorie-restricted diet (CRD), a high-protein diet (HPD), a low-carbohydrate diet, and a light-fasting diet. The primary goals in managing childhood obesity are reducing caloric intake and increasing physical activity while supporting healthy growth to lower body weight and promote lean mass. However, very-low-energy diet, low-carbohydrate, and light-fasting diets are often poorly tolerated in children because of their restrictive nature and uncertain long-term safety (9–11). A CRD remains the primary evidence-based option for managing childhood obesity (7, 8). However, prolonged energy restriction may limit adherence and provide inadequate protein and micronutrient intake, which may lead to impaired growth and muscle mass development (12).

In adults, an HPD has been shown to enhance satiety, preserve muscle mass, and reduce post-diet rebound (13, 14). However, in children, evidence of its effectiveness is limited, and its long-term use is not recommended. Short-term and intermittent HPD regimens have not been systematically evaluated in children. Recent research emphasizes the importance of multidisciplinary, short-term, and structured interventions that can achieve meaningful weight loss while preserving health and compliance in children (15, 16). However, challenges, such as poor adherence, high dropout rates, and rapid weight regain, persist, particularly in school-based programs. Therefore, approaches that combine effectiveness with sustainability are urgently required.

To address these challenges, we developed the “5 + 2” dietary management model, which combines 5 consecutive days of CRD followed by 2 days of HPD each week. The intervention is delivered under the supervision of pediatricians and registered dietitians and is supplemented with nutritional education, physical activity guidance, psychological support, and satiety-enhancing aids, such as high-fiber grain bars and gastric retention tablets. In this exploratory study, we aimed to evaluate the effectiveness of the “5 + 2” dietary management model in children with obesity. We assessed its effect on body weight, body composition, metabolic parameters, and behavioral outcomes after an 8-week intervention and a 12-week post-intervention follow-up period.

## 2 Methods

### 2.1 Study design and participants

This study was conducted as part of the study protocol “The family-school-healthcare collaborative plan for childhood obesity: protocol for a cluster-sampled interventional study,” based on the “Dietary intake, Regular exercise, Education, Assessment, and Monitoring” (DREAM) framework conducted in Shenzhen, China from October 1, 2023 to October 31, 2024 (17). Among the 115 children and adolescents with obesity enrolled in the DREAM program at the intervention school at baseline (October 2023), those who remained obese at the mid-term evaluation (March 2024) were screened for eligibility for the present study, which was conducted as a prospective non-randomized controlled trial between March and September 2024.

The inclusion criteria were: (1) age of 6–16 years; (2) obesity diagnosed according to the People’s Republic of China Health Industry Standard (18) at baseline in October 2023 and persisting in March 2024; and (3) provision of informed consent by the child and their parents.

The exclusion criteria were: (1) cognitive impairment or psychiatric disorders; (2) participation in other trials or use of medications affecting metabolism or weight loss within the past 3 months; or (3) other reasons making the child unsuitable for the study, based on clinical assessment performed by a physician.

**Figure 1:**
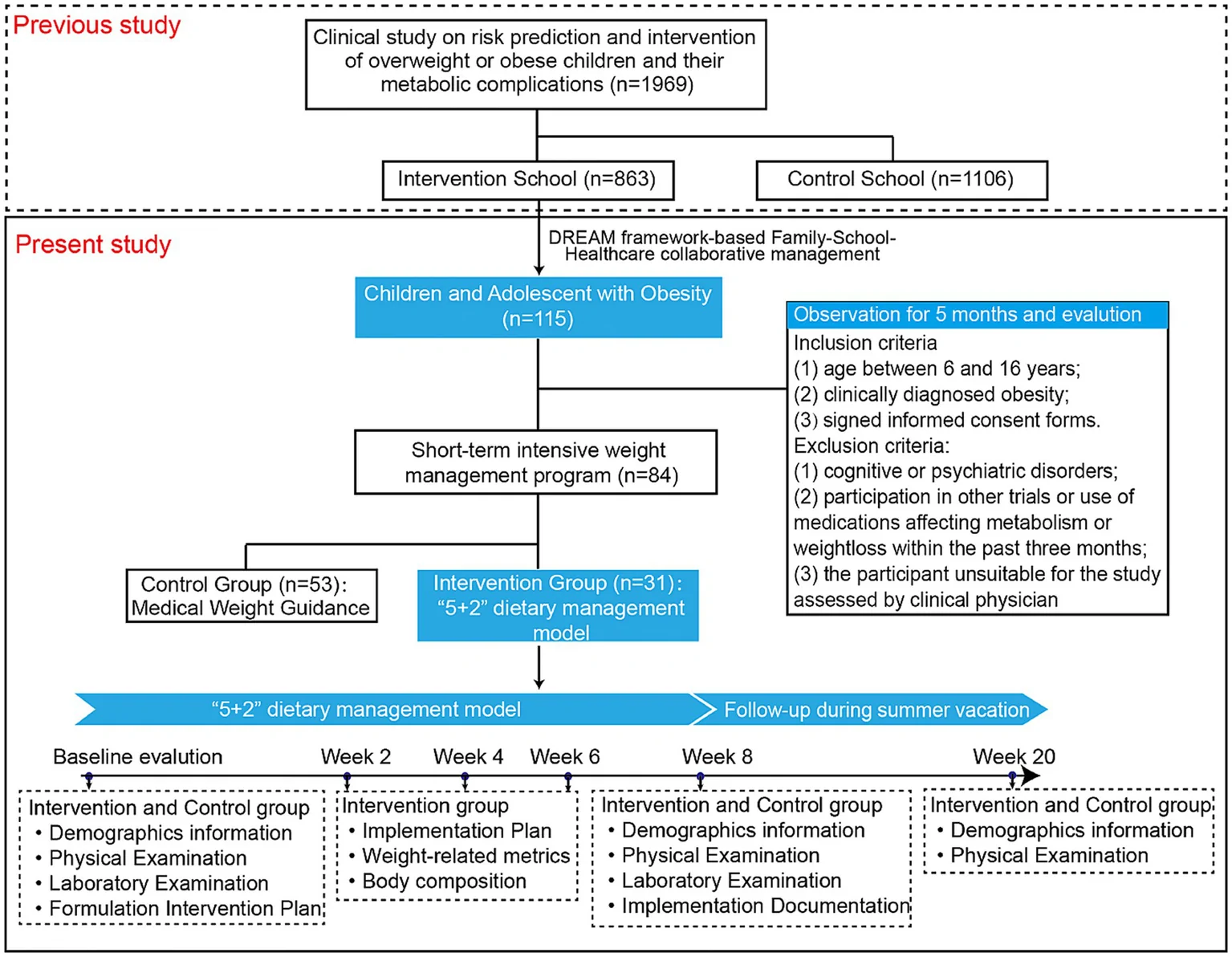
Flow chart of the research process. The flow chart outlines the recruitment process and the longitudinal design of the current study derived from a prior clinical investigation (*n* = 1,969). A total of 115 children and adolescents with obesity are identified via the DREAM framework-based collaborative management system. Following the application of specific inclusion and exclusion criteria, 84 participants are enrolled in a short-term intensive weight management program and allocated into either the Control Group (*n* = 53, receiving medical weight guidance) or the Intervention Group (*n* = 31, undergoing the “5 + 2” intermittent energy restriction dietary model). The timeline indicates the 8-week intensive intervention period followed by a follow-up assessment at week 20 during summer vacation. Evaluation metrics—including anthropometric measurements (physical examination), laboratory biochemical analysis, and body composition—were assessed at predefined intervals (baseline, week 2, 4, 6, 8, and 20) to monitor safety, compliance, and efficacy. DREAM, dietary intake, regular exercise, education, assessment, and monitoring.

A total of 84 participants met the eligibility criteria and were included in the present study. Participants who opted to take part in the intensive program were assigned to the intervention group, whereas those who did not were assigned to the control group. The intervention group received an 8-week structured “5 + 2” dietary intervention with behavioral, exercise, and sleep recommendations, along with scheduled behavior-rating assessments. The control group received conventional clinical weight-loss advice, including standard health education courses and physical education sessions, without additional structured dietary supervision, behavioral monitoring, or intervention programs. After the 8-week intervention, both groups entered a 12-week follow-up period that coincided with summer vacation in China, a period when children with obesity commonly regain weight after participating in weight-loss programs. The study flow chart is shown in Figure 1.

This study was approved by the Ethics Committee of Shenzhen Children’s Hospital (2024031) and conducted in accordance with the Declaration of Helsinki. Written informed consent was obtained from the participants and their parents. This study was registered in the Chinese Clinical Trial Registry (ChiCTR2400094705).

### 2.2 The “5 + 2” dietary management model

**Figure 2:**
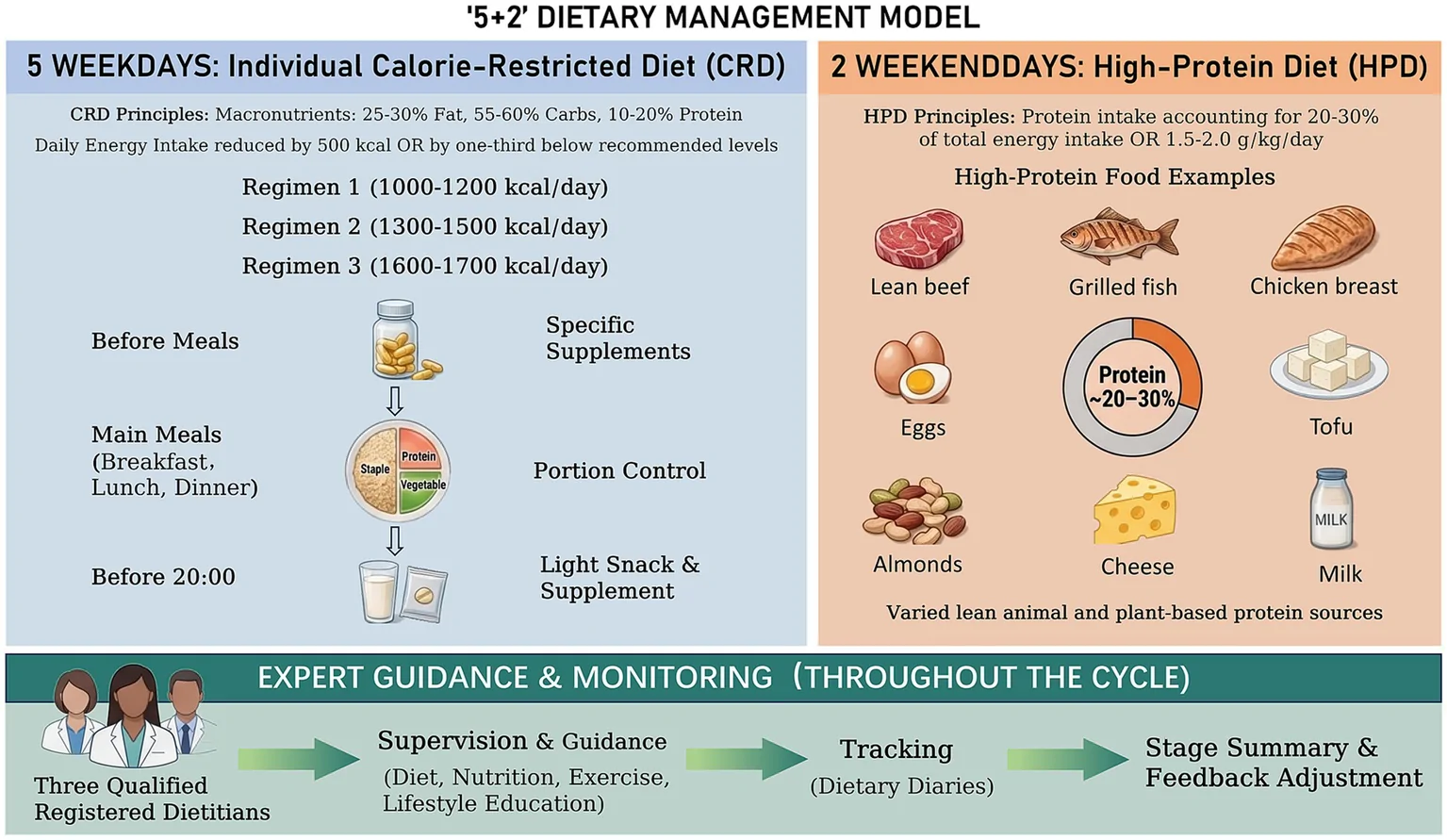
Schematic representation of the “5 + 2” dietary management model for childhood obesity intervention. The intervention protocol consists of two distinct phases tailored for children and adolescents with obesity. (Left) During the 5 weekdays, participants follow an individual calorie-restricted diet (CRD), characterized by a macronutrient distribution of 25–30% fat, 55–60% carbohydrate, and 10–20% protein. Daily energy intake is categorized into three regimens (1,000–1,700 kcal/day) based on age and physical activity level, with a focus on specific supplements and conventional foods. (Right) During the 2 weekend days, a high-protein diet (HPD) is implemented, with protein intake accounting for 20–30% of total energy (1.5–2.0 g/kg/day) using varied animal and plant-based sources. (Bottom) The entire cycle is supported by expert guidance from three qualified registered dietitians, encompassing continuous supervision, dietary diary tracking, and periodic feedback adjustments to ensure compliance and nutritional adequacy.

The “5 + 2” dietary management model was developed to address prevalent nutritional challenges among Chinese school-aged children, such as high caloric intake and irregular eating patterns (19, 20). This intervention integrated a 5-day school-based CRD with a 2-day home-based HPD on weekends (Figure 2).

For the CRD phase, each participant’s daily energy requirement (DER) was estimated individually based on age, sex, and physical activity level. The target energy intake was prescribed as DER minus 500 kcal/day (or a one-third reduction of the total DER) (7), maintaining a balanced macronutrient distribution of 55–60% carbohydrate, 25–30% fat, and 10–20% protein. To enhance satiety and alleviate dietary restriction-related distress, standardized nutritional supplements (e.g., nutrient composite slices and high-fiber cereal bars) were incorporated into the prescribed meal plans and consumed 15 min before or during main meals. Their energy contribution was included in the prescribed daily energy intake, and the nutritional composition of the standardized supplements is presented in Supplementary file 1.

Based on their individual calculated energy targets, participants were assigned to one of three energy-tiered regimens (Regimens 1–3). Specifically, Regimen 1 (1000–1,200 kcal/day) utilized nutrient composite slices and soluble fiber-enriched jelly to manage appetite at the lowest caloric tier. Regimen 2 (1300–1,500 kcal/day) focused on low-glycemic index management by incorporating high-fiber cereal bars, while Regimen 3 (1,600–1,700 kcal/day) prioritized satiety maintenance through the additional use of fiber-based gastric-occupying tablets. Detailed meal plans are provided in Supplementary file 2. Notably, all standardized nutritional supplements utilized in this study were food-grade, strictly adhering to the Food Safety Law of the People’s Republic of China and relevant national standards.

On weekends, participants followed a home-based HPD without meal replacements. Protein intake was prioritized at 20–30% of total energy or 1.5–2.0 g/kg of body weight per day (7).

Three registered dietitians with over 10 years of experience were stationed at the intervention school to supervise daily dietary implementation, provide nutrition and lifestyle education, monitor eating behaviors and satiety, review food diaries, and deliver individualized adjustments. Continuous guidance was delivered via a collaborative management platform to ensure protocol fidelity and promote sustainable behavioral change (Supplementary file 3).

### 2.3 Measurements

Baseline demographic and socioeconomic information, including parental educational level and annual household income, was collected. All participants underwent detailed physical examinations, body composition assessments, and biochemical analyses at baseline and at the end of the intervention period (week 8). At week 20, the weight-related metrics and body composition parameters were re-evaluated. The intervention group also underwent interim assessments at weeks 2, 4, and 6. During the intervention, detailed records of daily dietary intake, sleep, and physical activity levels were kept. Furthermore, personalized weight management behavior scores were collected at baseline and weeks 4 and 8. The score (0–100 points) was developed by our multidisciplinary pediatric obesity management team based on current pediatric obesity management guidelines and routine clinical practice to assess adherence to dietary, physical activity, and sleep recommendations during follow-up, with higher scores indicating better behavior management (Supplementary file 4).

Height and weight were measured using the Shanghe Ultrasonic Height and Weight Measuring Instrument (Model: SH-200, Zhengzhou Shanghe Electronic Technology company, Zhengzhou, China). The height standard deviation score (SDS), weight SDS, and BMI SDS were calculated by comparing individual measurements to age- and sex-specific reference values established in China (21, 22).

Body composition indices, including fat mass, muscle mass, and visceral fat area (VFA), were measured using a bioelectrical impedance analyzer (H-Key350; Beijing Seehigher Technology Co., Ltd., Beijing, China). Fat mass index (FMI) and muscle mass index (MMI) were calculated by dividing total fat mass and total muscle mass (kg), respectively, by height squared (m^2^), and are expressed as kg/m^2^. To minimize the influence of sex, age, and pubertal stage on body composition, FMI, MMI, and VFA were converted to sex-, age-, and pubertal stage-specific *Z*-scores using reference curves established from a population-based cohort of children aged 6–16 years in Shenzhen by our research group. Handgrip strength was measured using an electronic hand dynamometer (Jamar Plus+; Performance Health Supply Inc., Waukegan, IL, USA).

All participants had fasted for 10 h and fasting venous blood samples were collected in the morning. Fasting blood glucose, fasting insulin, triglyceride, total cholesterol, high-density lipoprotein cholesterol, low-density lipoprotein cholesterol, and uric acid levels were measured using automatic biochemical analyzer (model: Beckman Coulter K.K.AU5811, Beckman Coulter, America).

### 2.4 Outcomes

The primary outcomes were the change in weight-related metrics from weeks 0 to 8 and from weeks 8 to 20. The change in body composition parameters during the same intervals were the secondary outcomes. Additional outcomes included changes in behavioral habits and metabolic profiles.

### 2.5 Sample size

Sample size calculations were performed using power analysis and sample size software (NCSS Statistical Software, Kaysville, UT, USA). Based on prior interventional studies, the baseline mean BMI *Z*-score in the intervention and control groups was 0.7 and 0.8, respectively. The mean BMI *Z*-score after the intervention in the intervention group and in the control group was 0.4 and 0.7, respectively (23). The response rates were 60% in the intervention group and 20% in the control group. With 90% power, 5% significance, and a 1:1 allocation ratio, we calculated that 27 participants per group were required. Allowing for a 10% loss to follow-up, the required sample size was set at a minimum of 30 participants per group (60 in total). Therefore, the final study population of 84 participants exceeded the minimum required sample size, providing adequate statistical power for the primary analysis. A *post-hoc* power analysis based on the matched cohort demonstrated that the statistical power remained 84.5%.

### 2.6 Statistical analysis

The normality of continuous data was assessed using the Shapiro–Wilk test. Normally distributed continuous variables were presented as the mean ± standard deviation and compared using Welch two sample t-test. Non-normally distributed continuous variables were presented as median and quartiles and compared using the Wilcoxon rank sum test. Categorical variables were reported as counts (%) and compared using chi-squared tests or Fisher’s exact test. Propensity-score matching (PSM) based on age, sex, and baseline BMI was performed using 1:2 nearest-neighbor matching with a caliper value of 0.5 (Supplementary file 5). Covariate balance was evaluated using standardized mean differences. Comparisons within the matched dataset were conducted using t-tests. Sensitivity analyses were performed using the multivariate linear regression, adjusting for age, sex, and baseline BMI. Univariable linear regression analyses were performed within the intervention group to explore baseline and treatment characteristics associated with changes in BMI SDS at weeks 8 and 20. Regression coefficients (β) and corresponding 95% confidence intervals were reported. The trajectory of measurements was examined using repeated-measures analysis of variance with correction for the assumption of violations of sphericity. All tests were two-sided with α = 0.05. All analyses were performed using R version 4.1.2 (The R Foundation for Statistical Computing, Vienna, Austria).

## 3 Results

### 3.1 Baseline characteristics and PSM validation

The study included 84 participants (66 boys, 18 girls; median age 11.1 years; median BMI 24.8 kg/m^2^). Following PSM, the analysis was conducted on two balanced cohorts (*N* = 74; 30 in the intervention group and 44 in the control group). The baseline characteristics of the study population before and after PSM according to the two study groups are shown in Table 1.

**Table 1:** Baseline characteristics of the study population before and after propensity-score matching.

Characteristic	Missing (*n*)	Before PSM				After PSM			
		Overall *N* = 84	Intervention group *N* = 31	Control group *N* = 53	*p*	Overall *N* = 74	Intervention group *N* = 30	Control group *N* = 44	*p*
Sex, *n* (%)	0				0.42				0.683
Female		18 (21%)	5 (16%)	13 (25%)		14 (19%)	5 (17%)	9 (21%)	
Male		66 (79%)	26 (84%)	40 (76%)		60 (81%)	25 (83%)	35 (80%)	
Age (y)	0	11.1 (9.0, 12.1)	11.3 (10.6, 11.8)	10.4 (7.9, 12.4)	0.23	11.4 (9.6, 12.3)	11.4 (10.6, 11.8)	11.6 (9.1, 12.6)	0.956
Phase of puberty, n (%)	1				0.003				0.006
Pre-puberty		41 (49%)	10 (32%)	31 (60%)		34 (47%)	10 (33%)	24 (56%)	
Early puberty		34 (41%)	20 (65%)	14 (27%)		31 (43%)	19 (63%)	12 (28%)	
Late puberty		8 (10%)	1 (3%)	7 (14%)		8 (11%)	1 (3%)	7 (16%)	
Father’s Education	15				0.642				0.676
≤ High school		37 (53.6%)	17 (58.6%)	20 (50.0%)		32 (52.5%)	16 (57.1%)	16 (48.5%)	
> High school		32 (46.4%)	12 (41.4%)	20 (50.0%)		29 (47.5%)	12 (42.9%)	17 (51.5%)	
Mother’s education	15				0.222				0.164
≤ High school		43 (62.3%)	21 (72.4%)	22 (55.0%)		39 (63.9%)	21 (75.0%)	18 (54.5%)	
> High school		26 (37.7%)	8 (27.6%)	18 (45.0%)		22 (36.1%)	7 (25.0%)	15 (45.5%)	
Annual income	15				0.155				0.105
<120,000 RMB		42 (60.9%)	21 (72.4%)	21 (52.5%)		38 (62.3%)	21 (75.0%)	17 (51.5%)	
≥120,000 RMB		27 (39.1%)	8 (27.6%)	19 (47.5%)		23 (37.7%)	7 (25.0%)	16 (48.5%)	
Height (cm)	0	151.2 ± 14.4	153.9 ± 8.4	149.6 ± 16.8	0.12	153.3 ± 13.3	153.7 ± 8.4	153.1 ± 15.9	0.849
Height SDS	0	1.19 ± 0.92	1.25 ± 0.93	1.16 ± 0.91	0.71	1.15 ± 0.89	1.19 ± 0.88	1.13 ± 0.90	0.775
Weight (kg)	0	57.1 ± 15.7	60.6 ± 10.2	55.1 ± 18.0	0.081	59.6 ± 14.1	59.9 ± 9.7	59.4 ± 16.5	0.861
Weight SDS	0	2.69 (2.24, 3.14)	2.76 (2.03, 3.15)	2.67 (2.29, 3.12)	0.62	2.69 (2.25, 3.14)	2.72 (1.95, 3.13)	2.69 (2.34, 3.15)	0.706
BMI (kg/m^2^)	0	24.8 (22.6, 26.3)	25.2 (24.7, 26.5)	24.0 (21.8, 26.1)	0.027	25.0 (23.5, 26.4)	25.1 (24.7, 26.3)	24.7 (23.2, 26.5)	0.381
BMI SDS	0	2.17 (2.00, 2.43)	2.20 (2.04, 2.52)	2.15 (1.99, 2.38)	0.22	2.22 (2.03, 2.45)	2.17 (2.04, 2.47)	2.23 (2.02, 2.43)	0.823
Body fat mass (kg)	0	18.4 ± 5.49	20.4 ± 4.42	17.2 ± 5.74	0.006	19.3 ± 4.62	20.1 ± 4.22	18.7 ± 4.85	0.212
Body fat percentage (%)	0	32.1 ± 5.86	33.6 ± 4.86	31.2 ± 6.26	0.058	32.6 ± 5.85	33.5 ± 4.92	32.1 ± 6.39	0.271
FMI	0	7.91 ± 1.88	8.55 ± 1.56	7.54 ± 1.96	0.012	8.19 ± 1.70	8.47 ± 1.53	7.99 ± 1.81	0.225
FMI *Z*-score	9	3.27 ± 0.73	3.18 ± 0.59	3.32 ± 0.81	0.412	3.31 ± 0.75	3.18 ± 0.60	3.40 ± 0.84	0.261
Muscle mass (kg)	0	35.5 (27.7, 43.0)	37.0 (33.3, 42.0)	34.4 (25.4, 42.7)	0.130	35.9 (30.8, 43.8)	36.5 (33.2, 40.2)	35.7 (28.9, 45.4)	0.787
MMI	0	15.1 (14.1, 16.6)	15.4 (14.8, 17.1)	14.8 (13.9, 16.2)	0.130	15.3 (14.4, 16.8)	15.3 (14.7, 16.9)	15.2 (14.2, 16.7)	0.582
MMI *Z*-score		2.32 ± 1.21	2.41 ± 1.38	2.26 ± 1.10	0.636	2.44 ± 1.21	2.50 ± 1.33	2.39 ± 1.13	0.743
VAT (cm^2^)	0	81.9 ± 31.4	93.9 ± 27.1	74.8 ± 31.8	0.005	87.1 ± 27.3	92.7 ± 26.6	83.3 ± 27.4	0.147
VAT *Z*-score		4.11 ± 2.99	3.99 ± 1.04	4.19 ± 3.73	0.741	4.27 ± 3.13	3.96 ± 1.06	4.50 ± 4.05	0.478
BMD *Z*-score	0	−0.33 (−0.78, 0.35)	−0.52 (−0.91, 0.44)	−0.24 (−0.72, 0.27)	0.42	−0.44 (−0.87, 0.18)	−0.55 (−0.96, 0.50)	−0.35 (−0.79, 0.13)	0.783
Handgrip strength (kg)	0	16.8 (11.7, 21.8)	17.8 (15.5, 19.7)	15.7 (9.9, 22.6)	0.15	17.3 (13.2, 22.5)	17.9 (15.5, 19.7)	17.0 (11.5, 24.4)	0.787
Relative handgrip strength	0	0.65 (0.50, 0.82)	0.67 (0.60, 0.76)	0.63 (0.44, 0.86)	0.33	0.67 (0.54, 0.86)	0.67 (0.61, 0.78)	0.68 (0.49, 0.95)	0.969
Systolic BP (mmHg)	0	116 (108, 123)	119 (108, 123.5)	115 (108, 123)	0.84	118 (108.3, 123)	117.5 (108, 123.8)	118 (111, 123)	0.890
Diastolic BP (mmHg)	0	68 (63, 71)	69 (63, 71)	67 (63, 71)	0.51	68 (64, 71.8)	69 (62.5, 71)	68 (64, 72)	0.943
Triglyceride (mmol/L)	2	0.81 (0.61, 1.20)	0.99 (0.76, 1.29)	0.70 (0.59, 1.14)	0.016	0.88 (0.62, 1.28)	1.03 (0.78, 1.33)	0.73 (0.59, 1.27)	0.081
Total cholesterol (mmol/L)	2	4.41 (3.91, 5.07)	4.28 (3.85, 4.96)	4.46 (3.94, 5.12)	0.63	4.33 (3.85, 5.08)	4.29 (3.85, 5.02)	4.39 (3.84, 5.13)	0.950
HDL-C (mmol/L)	2	1.27 ± 0.22	1.21 ± 0.17	1.31 ± 0.24	0.036	1.26 ± 0.21	1.22 ± 0.17	1.29 ± 0.24	0.174
LDL-C (mmol/L)	2	3.01 (2.50, 3.57)	2.99 (2.55, 3.41)	3.01 (2.42, 3.75)	0.72	3.00 (2.49, 3.47)	2.99 (2.54, 3.44)	3.01 (2.32, 3.48)	0.591
Fasting glucose (mmol/L)	2	5.10 ± 0.34	5.17 ± 0.29	5.06 ± 0.37	0.23	5.11 ± 0.34	5.17 ± 0.29	5.06 ± 0.36	0.146
Fasting insulin (μU/mL)	3	12.18 ± 4.95	13.05 ± 4.15	11.64 ± 5.36	0.21	12.64 ± 4.98	13.17 ± 4.16	12.27 ± 5.51	0.435
HOMA-IR	3	2.79 ± 1.21	3.00 ± 0.99	2.66 ± 1.32	0.24	2.90 ± 1.23	3.03 ± 1.00	2.80 ± 1.37	0.412
Uric acid (μmol/L)	2	478 (362, 550)	496 (458.5, 552)	414 (346, 546)	0.049	489.5 (383.5, 556.3)	494.5 (450.3, 549.8)	469 (359568.5)	0.382

BMD, bone mineral density; BMI, body mass index; BP, blood pressure; FMI, fat mass index; HDL-C, high-density lipoprotein cholesterol; HOMA-IR, homeostasis model assessment of insulin resistance; LDL-C, low-density lipoprotein cholesterol; MMI, muscle mass index; PSM, propensity-score matching; SDS, standard deviation score; VAT, visceral adipose tissue.

### 3.2 Comparison of outcomes between the study groups

By the end of the 8-week intervention, the intervention group demonstrated significantly greater weight loss (−1.42 ± 1.73 kg vs. -0.44 ± 1.54 kg, *p* = 0.015) and a more pronounced reduction in BMI SDS (−0.21 ± 0.14 vs. -0.11 ± 0.14, *p* = 0.005) compared to the control group. The two groups showed no significant differences in the changes in fasting blood glucose, fasting insulin, triglyceride, total cholesterol, or uric acid levels from baseline to week 8. By the end of the follow-up period (week 20), the intervention group maintained a sustained reduction in body fat mass (−1.42 ± 3.41 kg), while the control group experienced significant fat regain (+1.84 ± 3.67 kg, *p* < 0.001). The intervention group achieved significantly higher skeletal muscle gain compared to controls (3.90 ± 3.46 kg vs. 2.21 ± 2.36 kg, *p* = 0.031). Consistent findings were observed using age-, sex-, and pubertal stage-adjusted body composition indices, with significantly lower FMI *Z*-scores and higher MMI *Z*-scores in the intervention group at week 20. Sensitivity analyses confirmed these findings (Table 2).

**Table 2:** Comparison of study outcomes.

Characteristic	Intervention group (*N* = 30)			Control group (*N* = 44)			Comparison and sensitivity analysis between the study groups					
	Baseline	Wk 8	Wk 20	Baseline	Wk 8	Wk 20	From baseline to wk 8			From wk 8 to wk 20		
							Difference (95% CI)	*p* ^a^	*p* ^b^	Difference (95% CI)	*p* ^a^	*p* ^b^
Height (cm)	153.68 ± 8.39	154.91 ± 8.47	156.26 ± 8.79	153.12 ± 15.85	154.08 ± 15.61	155.09 ± 14.53	0.22 (−0.03, 0.46)	0.085	0.156	0.34 (−0.08, 0.76)	0.112	0.185
Weight (kg)	59.93 ± 9.68	58.51 ± 9.30	62.46 ± 9.37	59.40 ± 16.53	58.95 ± 16.32	61.79 ± 16.75	−0.98 (−1.77, −0.19)	0.015	0.005	−0.87 (−2.03, 0.29)	0.140	0.052
BMI (kg/m^2^)	25.20 ± 2.06	24.23 ± 1.99	25.50 ± 1.98	24.73 ± 2.55	24.25 ± 2.58	25.35 ± 2.66	−0.49 (−0.79, −0.19)	0.002	0.001	−0.28 (−0.70, 0.15)	0.196	0.175
BMI SDS	2.28 ± 0.36	2.07 ± 0.45	2.33 ± 0.51	2.28 ± 0.45	2.17 ± 0.49	2.47 ± 0.54	−0.10 (−0.16, −0.03)	0.005	0.007	−0.07 (−0.17, 0.03)	0.152	0.311
Body fat mass (kg)	20.08 ± 4.22	18.58 ± 4.28	17.19 ± 4.32	18.74 ± 4.85	17.23 ± 4.92	18.90 ± 4.65	0.01 (−0.67, 0.70)	0.971	0.982	−3.26 (−5.00, −1.51)	<0.001	<0.001
Body fat percentage (%)	33.51 ± 4.92	31.75 ± 5.15	27.39 ± 4.89	32.05 ± 6.39	30.52 ± 6.30	31.30 ± 5.69	−0.23 (−1.72, 1.27)	0.761	0.979	−4.56 (−7.61, −1.51)	0.004	0.013
FMI	8.47 ± 1.53	7.73 ± 1.57	7.02 ± 1.49	7.99 ± 1.81	7.26 ± 1.83	7.91 ± 1.56	−0.01 (−0.28, 0.27)	0.961	0.989	−1.36 (−2.08, −0.63)	<0.001	0.002
FMI *Z*-score	3.18 ± 0.60	2.93 ± 0.72	2.50 ± 0.67	3.40 ± 0.84	3.04 ± 0.89	3.33 ± 0.91	0.05 (−0.09, 0.18)	0.508	0.710	−0.65 (−1.00, −0.31)	<0.001	<0.001
Muscle mass (kg)	37.58 ± 6.97	37.64 ± 6.74	41.71 ± 6.47	38.82 ± 13.41	39.30 ± 13.15	40.05 ± 12.75	−0.42 (−0.91, 0.07)	0.089	0.104	1.69 (0.17, 3.21)	0.031	0.041
MMI	15.78 ± 1.61	15.56 ± 1.46	17.02 ± 1.44	15.98 ± 2.40	16.01 ± 2.30	16.29 ± 2.27	−0.25 (−0.45, −0.05)	0.016	0.028	0.78 (0.13, 1.42)	0.020	0.033
MMI *Z*-score	2.50 ± 1.33	2.22 ± 1.29	3.41 ± 1.69	2.39 ± 1.13	2.41 ± 1.10	2.78 ± 1.56	−0.32 (−0.62, −0.02)	0.038	0.045	0.55 (−0.22, 1.33)	0.017	0.007
VAT (cm^2^)	92.66 ± 26.58	83.99 ± 27.40	—	83.31 ± 27.43	75.80 ± 25.87	—	−1.17 (−5.40, 3.07)	0.585	0.505	—	—	—
VAT *Z*-score	3.96 ± 1.06	3.60 ± 1.18	—	4.50 ± 4.05	3.76 ± 2.84	—	0.28 (−0.24, 0.79)	0.299	0.350	—	—	—
Handgrip strength (kg)	17.97 ± 4.00	20.26 ± 5.74	21.29 ± 5.57	20.02 ± 11.08	23.22 ± 13.18	22.58 ± 10.28	−0.92 (−2.62, 0.78)	0.284	0.319	−0.06 (−1.86, 1.73)	0.945	0.895
Relative handgrip strength	0.70 ± 0.14	0.84 ± 0.23	0.84 ± 0.23	0.77 ± 0.38	0.93 ± 0.47	0.88 ± 0.34	−0.02 (−0.09, 0.06)	0.621	0.734	0.01 (−0.07, 0.08)	0.808	0.771
Triglyceride (mmol/L)	1.06 ± 0.39	0.97 ± 0.37	—	0.96 ± 0.51	0.98 ± 0.38	—	−0.13 (−0.30, 0.03)	0.112	0.065	—	—	—
Total cholesterol (mmol/L)	4.47 ± 0.79	3.94 ± 0.54	—	4.52 ± 0.98	4.25 ± 0.86	—	−0.25 (−0.50, 0.00)	0.053	0.071	—	—	—
HDL-C (mmol/L)	1.22 ± 0.17	1.27 ± 0.19	—	1.29 ± 0.24	1.34 ± 0.28	—	0 (−0.09, 0.10)	0.931	0.708	—	—	—
LDL-C (mmol/L)	3.14 ± 0.76	2.07 ± 0.43	—	3.08 ± 1.03	2.25 ± 0.71	—	−0.25 (−0.51, 0.01)	0.062	0.071	—	—	—
Fasting glucose (mmol/L)	5.17 ± 0.29	4.77 ± 0.41	—	5.06 ± 0.36	4.77 ± 0.41	—	−0.08 (−0.27, 0.12)	0.445	0.285	—	—	—
Fasting insulin (μU/mL)	13.17 ± 4.16	12.09 ± 5.52	—	12.27 ± 5.51	10.21 ± 4.12	—	0.97 (−2.02, 3.96)	0.519	0.596	—	—	—
HOMA-IR	3.03 ± 1.00	2.61 ± 1.30	—	2.80 ± 1.37	2.17 ± 0.90	—	0.24 (−0.47, 0.95)	0.501	0.603	—	—	—
Uric acid (μmol/L)	498.17 ± 95.20	412.89 ± 99.81	—	481.07 ± 149.31	401.86 ± 117.32	—	−7.96 (−46.70, 30.78)	0.683	0.449	—	—	—

a *p*-value for between-group comparison in the matched cohort.

b *p*-value for sensitivity analysis.

BMI, body mass index; CI, confidence interval; FMI, fat mass index; HDL-C, high-density lipoprotein cholesterol; HOMA-IR, homeostasis model assessment of insulin resistance; LDL-C, low-density lipoprotein cholesterol; MMI, muscle mass index; SDS, standard deviation score; VAT, visceral adipose tissue.

### 3.3 Changes in the intervention group

In the intervention group, the weight-related metrics, including weight, BMI, waist circumference, and hip circumference, and body fat parameters, including body fat mass and visceral adipose tissue, decreased significantly over time (*p* < 0.01, Supplementary file 6). The median total behavior score significantly increased from 66.0 (59.0, 74.5) at baseline to 82.0 (75.5, 86.0) at week 8 (*p* < 0.001), indicating significant positive behavioral changes in the intervention group (Table 3). The proportion of participants who never weighed themselves dropped from 52 to 0% (*p* < 0.001). Participants meeting the target of >150 min of weekly exercise increased from 77 to 94% (*p* = 0.031).

**Table 3:** Behaviors in the intervention group at baseline and wk. 8 (*N* = 31).

Characteristic	Baseline	Wk 8	*p*
Weighing frequency^a^ (times/wk)			<0.001
Do not weigh	16 (52%)	0 (0%)	
2–3	11 (36%)	23 (74%)	
>5	4 (13%)	8 (26%)	
Breakfast habits			0.73
Eat breakfast 2–3 times/wk	4 (13%)	2 (6%)	
Finish breakfast before 9:00 a.m.	27 (87%)	29 (94%)	
Meal timing			0.82
Irregular 1–2 times/wk	7 (23%)	6 (19%)	
Regular mealtimes	24 (77%)	25 (81%)	
Dietary structure			0.83
Carbohydrates + protein (picky eater)	16 (52%)	15 (48%)	
Quality carbohydrates + quality protein + vegetables	14 (45%)	16 (52%)	
Only carbohydrates/long-term avoidance of carbohydrates	1 (3%)	0 (0%)	
Variety of food^a^ (types/d)			0.31
<6	1 (3%)	0 (0%)	
6–11	23 (74%)	23 (74%)	
≥12	7 (23%)	8 (26%)	
Eating speed^a^ (min)			< 0.001
<15 or >40	14 (45%)	2 (6%)	
15–20	13 (42%)	25 (81%)	
20–30	4 (13%)	4 (13%)	
Eating order			< 0.001
Protein, staple food, vegetables/soup	7 (23%)	11 (37%)	
Soup, vegetables, protein, staple food	6 (19%)	18 (60%)	
Staple food, protein, vegetables/soup	18 (58%)	1 (3%)	
Unknown	0	1	
Satiety level^a^			< 0.001
Overly full/overly hungry	6 (20%)	0 (0%)	
90% full	15 (50%)	0 (0%)	
70–80% full	9 (30%)	30 (100%)	
Unknown	1	1	
Cooking methods			0.52
Mainly stir-fried, stewed, braised, or marinated	15 (48%)	17 (55%)	
Mainly sweet and sour, dry-fried, red-cooked, or deep-fried	11 (36%)	7 (23%)	
Mainly steamed, boiled, blanched, cold-mixed, or water–oil braised	5 (16%)	7 (23%)	
Frequency of dining out (times/wk)			>0.9
1–2	29 (94%)	29 (94%)	
3–6	2 (6%)	2 (6%)	
Frequency of binge eating/emotional eating^a^ (times/mo)			0.048
≥3	6 (20%)	1 (3%)	
1–2	11 (37%)	16 (52%)	
Do not binge eat	13 (43%)	14 (45%)	
Unknown	1	0	
Frequency of snacks/tea drinks/sweets/fried foods^a^			0.073
≥3 times/wk	10 (32%)	3 (10%)	
1–2 times/wk	9 (29%)	16 (53%)	
1–2 times/mo	12 (39%)	11 (37%)	
Unknown	0	1	
Frequency of afternoon tea/late-night snack^a^			0.011
≥3 times/wk	9 (29%)	3 (10%)	
1–2 times/wk	9 (29%)	13 (43%)	
1–2 times/mo	13 (42%)	14 (47%)	
Unknown	0	1	
Water intake^a^ (mL)			0.005
<1,000	8 (26%)	1 (3%)	
1,000–1,500	8 (26%)	12 (39%)	
1,500–1700	15 (48%)	18 (58%)	
Exercise frequency^a^ (times/wk)			0.24
Sedentary/no exercise habits	3 (10%)	0 (0%)	
1–2	1 (3%)	2 (6%)	
≥3	27 (87%)	29 (94%)	
Weekly total exercise duration^a^ (min/wk)			0.031
<30	3 (10%)	0 (0%)	
60–120	4 (13%)	2 (6%)	
>150	24 (77%)	29 (94%)	
Heart rate after exercise			0.41
Slow (<100 beats/min)	5 (16%)	2 (6%)	
Moderately elevated (100–140 beats/min)	26 (84%)	29 (94%)	
Respiratory rate after exercise			0.62
Rapid	10 (32%)	11 (36%)	
Relatively rapid	14 (45%)	16 (52%)	
Steady	7 (23%)	4 (13%)	
Sleep onset time^a^			0.82
After 12:00 a.m.	2 (6%)	0 (0%)	
Between 10:00 p.m. and 12:00 a.m.	17 (55%)	22 (71%)	
Before 10:00 p.m.	12 (39%)	9 (29%)	
Sleep duration (h)			> 0.9
≤7 or ≥12	1 (3%)	0 (0%)	
7–9	23 (74%)	24 (77%)	
8–10	1 (3%)	1 (3%)	
9–11	6 (19%)	6 (19%)	
Total behavior score^a^	66.0 (59.0, 74.5)	82.0 (75.5, 86.0)	<0.001

a Compared using paired Wilcoxon rank-sum test.

### 3.4 Key factors influencing BMI SDS reduction

In the linear regression analysis, sex, age, adherence, and intervention were identified as the primary drivers of changes in the BMI SDS (Table 4). At week 8, the reduction in the BMI SDS was significantly greater in boys than in girls (*β* = −0.758, *p* = 0.015), older children than in younger children (*β* = −0.240, *p* = 0.003) and children with higher baseline adherence than in those with lower adherence (*β* = −0.356, *p* = 0.004). At week 20, the reductions in the BMI SDS was significantly greater in those who followed regimen 3 (*β* = −1.702, *p* = 0.049) and those who ate staple food first (*β* = −4.193, *p* = 0.006).

**Table 4:** Association between baseline and treatment characteristics and changes in body mass index standard deviation score.

Characteristic	Intervention group *N* = 31	Wk 8		Wk 20	
		Coefficient (95% CI)	*p*	Coefficient (95% CI)	*p*
Sex, *n* (%)					
Female	5 (16%)	Reference		Reference	
Male	26 (84%)	−0.758 (−1.360, −0.157)	0.015	−0.337 (−1.174, 0.499)	0.415
Age (y)	11.3 (10.6, 11.8)	−0.240 (−0.390, −0.089)	0.003	−0.202 (−0.422, 0.017)	0.069
Student type, *n* (%)					
Boarding	19 (61%)	Reference		Reference	
Non-boarding	12 (39%)	0.090 (−0.413, 0.592)	0.717	−0.080 (−0.729, 0.569)	0.801
Baseline characteristics					
Child adherence score	4.0 (3.0, 4.5)	−0.356 (−0.585, −0.127)	0.004	−0.049 (−0.397, 0.298)	0.773
Parental support score	3.0 (3.0, 4.0)	−0.140 (−0.353, 0.073)	0.189	−0.019 (−0.330, 0.291)	0.899
Total behavior score	66.0 (59.0, 74.5)	0.008 (−0.012, 0.027)	0.417	0.003 (−0.023, 0.028)	0.816
Intervention characteristics					
Intervention regimen calories (kcal/d), *n* (%)					
1,100–1,400	17 (55%)	Reference		Reference	
1,500–1,600	14 (45%)	−0.165 (−0.654, 0.324)	0.495	−0.278 (−0.912, 0.356)	0.376
Intervention regimen, *n* (%)					
Regimen 1	1 (3%)	Reference		Reference	
Regimen 2	16 (52%)	−0.662 (−2.062, 0.739)	0.341	−1.519 (−3.201, 0.163)	0.075
Regimen 3	14 (45%)	−0.788 (−2.194, 0.618)	0.261	−1.702 (−3.393, −0.012)	0.049
Dietary fibre jelly consumption (d)	25.0 (15.0, 29.5)	−0.012 (−0.031, 0.007)	0.216	−0.006 (−0.031, 0.018)	0.612
Behavior during the intervention period					
Change in total behavior score	13.0 (8.5, 17.5)	0.008 (−0.012, 0.027)	0.417	0.003 (−0.023, 0.028)	0.816
Weighing frequency >5 times/wk. at wk. 8, *n* (%)	8 (26%)	0.166 (−0.226, 0.557)	0.394	0.273 (−0.276, 0.821)	0.317
Proportion of days consuming soup first	0.53 (0.40, 0.58)	1.142 (−0.591, 2.876)	0.188	1.842 (−0.340, 4.023)	0.095
Proportion of days consuming vegetables first	0.24 (0.10, 0.34)	−0.512 (−2.086, 1.063)	0.512	0.138 (−1.982, 2.259)	0.894
Proportion of days consuming meat first	0.17 (0.07, 0.21)	0.010 (−2.386, 2.407)	0.993	0.031 (−3.118, 3.179)	0.984
Proportion of days consuming staple foods first	0.06 (0.05, 0.17)	−0.998 (−3.497, 1.501)	0.421	−4.193 (−7.062, −1.324)	0.006
Average meal duration (min)	12.0 (11.5, 12.8)	−0.075 (−0.334, 0.183)	0.556	−0.130 (−0.456, 0.195)	0.418
Average daily water intake (mL)	1,220 (1,032, 1,329)	−0.000 (−0.001, 0.000)	0.309	0.000 (−0.001, 0.002)	0.572
Average daily exercise duration (min)	52.0 (48.3, 57.0)	−0.015 (−0.047, 0.017)	0.337	−0.003 (−0.044, 0.038)	0.886

CI, confidence interval.

## 4 Discussion

This exploratory prospective controlled study demonstrated that the structured “5 + 2” dietary management model was associated with favorable changes in body composition and health-related behaviors among children and adolescents with obesity. Compared with conventional weight management, participants receiving the intervention showed greater reductions in BMI SDS together with greater skeletal muscle gain and less fat regain after the summer vacation. These findings suggest that integrating structured dietary modification with behavioral support may provide a practical strategy for improving short-term obesity management in pediatric populations.

The favorable outcomes observed in the present study may reflect the complementary physiological effects of weekday calorie restriction and weekend moderate high-protein intake. The calorie-restricted phase created a sustained negative energy balance while fitting well within regular school routines, which may have facilitated adherence. Meanwhile, moderate protein enrichment has been reported to enhance satiety through increased secretion of appetite-regulating gastrointestinal hormones, including glucagon-like peptide-1, cholecystokinin and peptide YY, thereby activating central satiety pathways and suppressing hunger (24, 25). Higher protein intake has also been associated with preservation of lean body mass through stimulation of muscle protein synthesis and reduced proteolysis (26, 27). In addition, the structured meal plan addressed common obesogenic behaviors, such as snacking and late-night eating, by providing practical dietary alternatives, including high-fiber bars, which may have facilitated healthier eating habits (28, 29).

Current evidence regarding high-protein diets in children remains limited. A recent meta-analysis suggested that short-term high-protein dietary interventions may improve BMI in children with overweight or obesity, whereas no consistent benefits were observed for body fat percentage or blood lipid profiles (14). Our findings are broadly consistent with this evidence while suggesting that the observed improvements may not be attributable to protein intake alone. Rather, the combination of moderate intermittent protein enrichment with calorie restriction, behavioral counseling, exercise recommendations, and regular follow-up may have contributed to the favorable changes in body composition. Importantly, protein intake in our protocol was restricted to 2 days per week and remained within recommended pediatric intake ranges. Although concerns have been raised regarding the long-term safety of high-protein diets in growing children, only three of the eight studies included in the above meta-analysis reported safety outcomes, and none identified intervention-related adverse events (30–32). Similarly, no adverse events or deterioration in biochemical indicators were observed during the present study. Nevertheless, because the follow-up period was relatively short, future studies with dedicated long-term monitoring of renal function, bone metabolism, and growth are required to further establish the safety of this dietary approach.

Recent pediatric studies have also demonstrated the feasibility of intermittent energy restriction for obesity management. A multicenter randomized clinical trial comparing intermittent energy restriction with continuous energy restriction in adolescents with obesity reported similar reductions in BMI SDS after 52 weeks, with no significant between-group differences in body composition (33). Likewise, another prospective study using a cyclic intermittent energy restriction regimen consisting of three very-low-energy days and four healthy eating days each week demonstrated significant improvements in BMI, body fat percentage, eating behaviors, and quality of life over 26 weeks (34). Consistent with these findings, our structured “5 + 2” dietary model was associated with improvements in BMI SDS and health-related behaviors. In addition, we observed greater skeletal muscle gain and less fat regain during follow-up. Importantly, these favorable body composition changes remained evident after adjustment for age, sex, and pubertal stage. These findings suggest that combining moderate intermittent protein enrichment with calorie restriction, together with structured behavioral support and regular follow-up, may provide additional benefits for body composition beyond intermittent energy restriction alone.

The behavioral findings provide an important complementary explanation for the observed clinical outcomes. Participants in the intervention group demonstrated greater improvements in self-monitoring behaviors, particularly regular self-weighing and physical activity. The structured weekly cycle of the “5 + 2” model, together with scheduled behavioral assessment and feedback, may have facilitated habit formation by providing clear and predictable behavioral targets. Regular self-monitoring has consistently been associated with improved adherence and better weight-management outcomes in pediatric obesity interventions (35). In our study, the proportion of participants achieving at least 150 min of weekly exercise also increased significantly, which may have further contributed to the favorable changes in body composition.

Multiple individual-level factors may influence adherence to obesity interventions. In our cohort, older children experienced greater reductions in BMI SDS at week 8. Several factors may contribute to this association, including better understanding of behavioral recommendations, greater autonomy in implementing lifestyle changes, or developmental differences in energy expenditure (36). However, these potential explanations were not directly evaluated in the present study and should therefore be interpreted cautiously. Consistent with previous evidence demonstrating dose–response relationships in childhood obesity interventions (37), higher adherence scores were associated with better short-term outcomes in our study, further emphasizing the importance of sustained participant engagement. The relatively large variability observed in the control group may also reflect individual differences in adherence to conventional weight-management advice, particularly during the summer vacation when daily routines and lifestyle behaviors were less structured.

This study has several strengths. First, the intervention integrated dietary management, behavioral counseling, and exercise guidance within a structured program, reflecting current multidisciplinary recommendations for pediatric obesity management. Second, the prospective controlled design with repeated follow-up assessments enabled evaluation of both immediate intervention effects and short-term maintenance after the summer vacation. Third, assessment of body composition, together with age-, sex-, and pubertal stage-standardized body composition indices, provided a more comprehensive evaluation of intervention-related physiological changes while minimizing the influence of normal growth and pubertal development.

Several limitations should be acknowledged. First, the prospective non-randomized design may have introduced selection bias and residual confounding. Although PSM improved baseline comparability between groups, these approaches cannot fully eliminate bias inherent to a non-randomized study. Therefore, the observed associations should be interpreted with caution. Second, the relatively modest sample size may have limited statistical power. Finally, this was a single-center study with a relatively short follow-up period. Future multicenter randomized controlled trials with larger sample sizes and longer follow-up are warranted to confirm these findings and further evaluate the long-term efficacy and safety of this dietary approach.

## 5 Conclusion

The “5 + 2” dietary management model was associated with favorable changes in body composition and health-related behaviors among children and adolescents with obesity. These findings suggest that this structured dietary approach may represent a practical option for pediatric obesity management in clinical settings where appropriate dietary guidance and follow-up can be provided. Future randomized controlled trials with longer follow-up are warranted to confirm these findings and evaluate their long-term sustainability.

## Data Availability

The original contributions presented in the study are included in the article/Supplementary material, further inquiries can be directed to the corresponding author.
