# Supplementary material for: Effects of a “5 + 2” dietary management model on body composition and behavioral modification in Chinese children with obesity: a prospective, non-randomized controlled trial
Source: Front Nutr. 2026 Sep 1;13:1907795. doi: 10.3389/fnut.2026.1907795 (PMC13574584; doi:10.3389/fnut.2026.1907795)
Supplement: Supplementary file 1 [file Table_1.DOCX]

**Additional files**

**Additional file 1.** Nutritional composition of the standardized food-grade supplements (per 100g)

| **Supplement Name** | **Energy**  **(KJ)** | **Protein**  **(g)** | **Fat**  **(g)** | **Carbohydrate**  **(g)** | **Dietary Fiber**  **(g)** | **Sodium**  **(mg)** |
| --- | --- | --- | --- | --- | --- | --- |
| Nutrient composite slices | 1532 | 2.1 | 0.4 | 87.1 | - | 2.1 |
| High-fiber cereal bar | 1503 | 19.4 | 12.5 | 35.9 | 12.5 | 221.0 |
| Fiber-based gastric-occupying tablets | 791 | 0 | 0 | 23.0 | 50.0 | 50.0 |
| Soluble fiber-enriched jelly | 448 | 0 | 0 | 17.2 | 19.5 | 93.0 |
| Probiotic powder | 1312 | 4.1 | 0 | 58.5 | 30.1 | 60 |

**Additional file 2.** Detailed meal plans for the three calorie-restricted regimens

| **Regimen** | **Meal** | **Detailed meal compositions** |
| --- | --- | --- |
| Regimen 1 (1000–1200 kcal/day) | Breakfast | 0.5 portion of staple food, 1 portion of soy milk or milk, 1 egg, and 1 portion of vegetables. |
|  | Lunch | 2 packets of nutrient composite slices (15 minutes before or with meal); 0.5 portion of staple food, 1 portion of meat/egg/tofu, and 1.5–2 portions of vegetables. |
|  | Dinner | 2 packets of nutrient composite slices (15 minutes before or with the meal); 0.5 portion of staple food, 1 portion of meat/egg/tofu, and 1.5–2 portions of vegetables. |
|  | Before 20:00 | 1 packet of soluble fiber-enriched jelly, and 1 packet of probiotic powder; 1 portion of milk. |
| Regimen 2 (1300–1500 kcal/day) | Breakfast | 2 packets of nutrient composite slices (15 minutes before or with meal); 1 portion of staple food, 1 portion of soy milk or milk, 1 egg, and 1 portion of vegetables. |
|  | Lunch | 2 packets of nutrient composite slices (15 minutes before or with meal); 1 portion of staple food, 1–1.5 portions of meat/egg/tofu, and 1.5–2 portions of vegetables. |
|  | Dinner | 1 packet of high-fiber cereal bar (15 minutes before or with meal); 1 portion of staple food, 1–1.5 portions of meat/egg/tofu, and 1.5–2 portions of vegetables. |
|  | Before 20:00 | 1 packet of probiotic powder and 1 portion of milk. |
| Regimen 3 (1600–1700 kcal/day) | Breakfast | 2 packets of nutrient composite slices (15 minutes before or with meal); 1 portion of staple food, 1 portion of soy milk or milk, 1 egg, and 1 portion of vegetables. |
|  | Lunch | 3–4 fiber-based gastric-occupying tablets (15 minutes before the meal with 200 ml water); 1–1.5 portions of staple food, 2 portions of meat/egg/tofu, and 2 portions of vegetables. |
|  | Dinner | 1 packet of high-fiber cereal bar (15 minutes before or with meal); 1 portion of staple food, 2 portions of meat/egg/tofu, and 2 portions of vegetables. |
|  | Before 20:00 | 3–4 fiber-based gastric-occupying tablets and 1 portion of milk. |

**Additional file 3.** Table used by dietitians to record relevant intervention measures and dietary conditions

| **Daily Check-in Record** | | | | | | | |
| --- | --- | --- | --- | --- | --- | --- | --- |
| 1. Date: | ______ Month  ______ Day (Mon) | ______ Month  ______ Day (Tue) | ______ Month  _____ Day (Wed) | ______ Month  ______ Day (Thu) | ______ Month   ______ Day (Fri) | _____ Month  ______ Day (Sat) | ______ Month   ______ Day (Sun) |
| 2. Yesterday's Bowel Movement: |  |  |  |  |  |  |  |
| 3. Today's Exercise Log:  (Include type & duration of exercise) |  |  |  |  |  |  |  |
| 4.Diet Log:  Breakfast: | Carbs: ______ fists  Protein: ______ palms  Vegetables: _____palms | Carbs: ______ fists  Protein: ______ palms  Vegetables: _____palms | Carbs: ______ fists  Protein: _____ palms  Vegetables: ____palms | Carbs: ______ fists  Protein: ______ palms  Vegetables: _____palms | Carbs: ______ fists  Protein: ______ palms  Vegetables: _____palms | Carbs: ______ fists  Protein: ______ palms  Vegetables: _____palms | Carbs: ______ fists  Protein: ______ palms  Vegetables: ____ palms |
| Lunch: | Carbs: ______ fists  Protein: ______ palms  Vegetables: ____ palms | Carbs: ______ fists  Protein: ______ palms  Vegetables: ____ palms | Carbs: ______ fists  Protein: ______ palms  Vegetables: ___ palms | Carbs: ______ fists  Protein: ______ palms  Vegetables: ____ palms | Carbs: ______ fists  Protein: ______ palms  Vegetables: ____ palms | Carbs: ______ fists  Protein: ______ palms  Vegetables: ____ palms | Carbs: ______ fists  Protein: ______ palms  Vegetables: ____ palms |
| Dinner: | Carbs: ______ fists  Protein: ______ palms  Vegetables: ____ palms | Carbs: ______ fists  Protein: ______ palms  Vegetables: ____ palms | Carbs: ______ fists  Protein: ______ palms  Vegetables: ____ palms | Carbs: ______ fists  Protein: ______ palms  Vegetables: ____ palms | Carbs: ______ fists  Protein: ______ palms  Vegetables: _____palms | Carbs: ______ fists  Protein: ______ palms  Vegetables: ____ palms | Carbs: ______ fists  Protein: ______ palms  Vegetables: ____ palms |
| Snacks:  (Time, type & quantity) |  |  |  |  |  |  |  |

| 5. Water Intake:  (Recommended: 1500-1700ml) | ______ ml | ______ ml | ______ ml | ______ ml | ______ ml | ______ ml | ______ ml |
| --- | --- | --- | --- | --- | --- | --- | --- |
| 6. Eating Order:  (Recommended: Soup → Vegetables → Protein → Carbs) |  |  |  |  |  |  |  |
| 7. Eating Speed:  (Recommended: 20 chews per bite, 15-20 min per meal) | ______ minutes | ______ minutes | ______ minutes | ______ minutes | ______ minutes | ______ minutes | ______ minutes |
| 8. Fullness Level?  (Recommended: 7-8/10 full) | □ 7/10 full  □ 8/10 full  □ 9/10 full  □ 10/10 full | □ 7/10 full  □ 8/10 full  □ 9/10 full  □ 10/10 full | □ 7/10 full  □ 8/10 full  □ 9/10 full  □ 10/10 full | □ 7/10 full  □ 8/10 full  □ 9/10 full  □ 10/10 full | □ 7/10 full  □ 8/10 full  □ 9/10 full  □ 10/10 full | □ 7/10 full  □ 8/10 full  □ 9/10 full  □ 10/10 full | □ 7/10 full  □ 8/10 full  □ 9/10 full  □ 10/10 full |
| 9. Grain Bars | ______ bars/day | ______ bars/day | ______ bars/day | ______ bars/day | ______ bars/day | ______ bars/day | ______ bars/day |
| 10. Urine Ketones: |  |  |  |  |  |  |  |
| 11. Last Night's Bedtime:  (Recommended: 9:30 PM) |  |  |  |  |  |  |  |
| 12. Personal Notes: (Discomfort/hunger/low mood, etc.) |  |  |  |  |  |  |  |

**Additional file 4.** Personalised one-on-one medical weight management behaviour scoring sheet.

| □Before Weight Loss □After 1 Month □After 2 Months □After 3 Months | | | | | |
| --- | --- | --- | --- | --- | --- |
|  | Item | 5 Points | 3 Points | 1 Point | Score |
| Dietary habits and behaviors 70 points | Measure body weight | >5 times/week | 2-3 times/week | Never weigh |  |
|  | Breakfast | Eat breakfast before  9:00 AM daily | Eat breakfast 2-3 times/week | Skip breakfast |  |
|  | Meal Timing | Regular meals | Irregular 1-2 times/week | Highly irregular meals |  |
|  | Unbalanced Diet | Quality carbs + protein + vegetables | Carbs + protein (picky eating) | Only carbs/long-term no carbs |  |
|  | Food Variety | ≥12 types/day | 6-11 types/day | <6 types/day |  |
|  | Eating Speed | 20-30 minutes | 15-20 minutes | <15 min or >40 min |  |
|  | Meal Order | Soup-vegetables-protein-carbs | Protein-carbs-vegetables/soup | Carbs-protein-vegetables/soup |  |
|  | Fullness Level | 7-8/10 full | 9/10 full | Overeating/extreme hunger |  |
|  | Cooking Methods | Steaming, boiling, stir-frying, cold dishes, water-oil sautéing | Light stir-frying, stewing, braising, marinating | Sweet-sour, dry-frying, red-braising, deep-frying |  |
|  | Eating Out Frequency | 1-2 times/week | 3-6 times/week | 7-13 times/week or >2 times/day |  |
|  | Binge eating  / Frequency of emotional eating | No binge eats | 1-2 times/month | ≥3 times/month |  |
|  | Frequency of consuming high-fat and high-sugar snacks and beverages | 1-2 times/month | 1-2 times/week | ≥3 times/week |  |
|  | Frequency of afternoon tea or late-night snacks | 1-2 times/month | 1-2 times/week | ≥3 times/week |  |
|  | Water Intake | 1500-1700ml | 1000-1500ml | <1000ml |  |
| Exercise  20 points | Exercise Frequency | ≥3 times/week | 1-2 times/week | Sedentary/no exercise habit |  |
|  | Weekly Exercise Duration | ≥150 minutes | 60-120 minutes | <30 minutes |  |
|  | Post-Exercise Heart Rate | 100-140 bpm | >140 bpm | <100 bpm |  |
|  | Post-Exercise Feeling | Moderately breathless | Very breathless | Normal breathing |  |
| Sleep 10 points | Bedtime | Before 22:00 | 22:00-24:00 | After 24:00 |  |
|  | Sleep Duration (6-13 yrs) | 9-11 hours | 7-9 hours | ≤7 or ≥12 hours |  |
|  | Sleep Duration (14-17 yrs) | 8-10 hours | 6-8 hours | ≤6 or ≥12 hours |  |
|  | Sleep Duration (Adults) | 7-9 hours | 5-7 hours | ≤4 or ≥12 hours |  |
| <60 points - Poor behavior habits, 60-80 points - Moderate behavior habits, >80 points - good behavior habits | | | | |  |

**Additional file 5.** Summary statistics for propensity score matching

| **Characteristic** | | **SMD** | **Variance ratio** | **eCDF (Mean)** | **eCDF (Max)** |
| --- | --- | --- | --- | --- | --- |
| Before PSM | Distance | 0.581 | 0.835 | 0.156 | 0.292 |
|  | Age (years) | 0.464 | 0.312 | 0.156 | 0.310 |
|  | Sex | 0.228 |  | 0.084 | 0.084 |
|  | BMI (kg/m^2^) | 0.699 | 0.502 | 0.142 | 0.391 |
| After PSM | Distance | 0.130 | 1.027 | 0.048 | 0.167 |
|  | Age (years) | -0.039 | 0.390 | 0.108 | 0.217 |
|  | Sex | 0.091 |  | 0.033 | 0.033 |
|  | BMI (kg/m^2^) | 0.108 | 0.762 | 0.051 | 0.267 |

SMD, standardized mean difference; eCDF, empirical cumulative distribution function; PSM, propensity score matching; BMI, body mass index.

**Additional file 6.** Trajectories of weight-related metrics and body composition parameters in the intervention group.

Blue points represent the mean values. *P*-values were derived from repeated measures ANOVA analysis.

BMI, body mass index; VAT, visceral adipose tissue.
